# The inhibition of PARG attenuates DNA repair in hepatocellular carcinoma

**DOI:** 10.1186/s43556-023-00114-6

**Published:** 2023-01-31

**Authors:** Longpo Geng, Yaling Sun, Mingming Zhu, Hongda An, Yunzheng Li, Yuanxiang Lao, Yongli Zhang, Binghua Li, Jie Ni, Zhu Xu

**Affiliations:** 1 Department of Hepatobiliary Surgery, The Affiliated Drum Tower Hospital of Nanjing University Medical School, Nanjing, 210008 China; 2 Department of Radiation Oncology, The Third Affiliated Hospital of Sun Yat-Sen University, Guangzhou, 510630 China; 3 Department of Gynecology of Shanghai First Maternity & Infant Hospital, Tongji University School of Medicine, Shanghai, 201204 China

To the editor,

Poly(ADP-ribose) polymerase 1 (PARP1), a most abundant member of the Poly(ADP‐ribose) polymerase (PARP) superfamily, autoPARylates itself and PARylates a huge number of target proteins in response to DNA single-stranded breaks (SSB) or DNA double-stranded breaks (DSBs), such as BRCA1 [1], the Ku complex [2]. Poly(ADP-ribosyl)ation (PARylation), composed of linear or branched chains of ADP-ribose units, is a unique and transient posttranslational modification for preserving the genome integrity by using nicotinamide adenine dinucleotide (NAD^+^) as the donor. While PARylation plays an important role in DNA damage repair, the excessive PARylation generated by PARP1 will cause cell death via the pathway of parthanatos, a unique form of caspase-independent programmed cell death. Poly(ADP-ribose) glycohydrolase (PARG) is the only enzyme responsible for hydrolyzing poly(ADP-ribose) (PAR) chain which is produced by PARP1. It has been reported that PARG could be recruited to DSBs site in a PARP1 and PCNA-dependent manner in Hela cells using laser micro irradiation [3], which clarified the importance of PARG in DSBs repair. The inhibition of PARG with COH34 impaired DNA repair by prolonging PARylation at DNA lesions and trapping DNA repair factors and significantly suppressed DNA repair-defective and PARP inhibitor-resistant cancers [4]. PARG deficient Hela cells show increased sensitivity to γ irradiation and a delay in the repair of X-Ray induced DSBs [4]. Although the important role of PARG in DNA damage repair has been extensively studied in different cancer cells, the functional significance of PARG in hepatocellular carcinoma (HCC) still need to be further explored, especially DSBs.

Liver cancer remains a highly life-threatening cancer and has long been a worldwide public health concern. HCC accounts for approximately 90% of primary liver cancer cases. The poor 5-year survival rate (less than 20%) and prognosis highlight the urgent need to identify new therapeutic interventions for the treatment of this disease. DSBs are the most dangerous forms of DNA lesions among various types of DNA damage, such as SSB, base damage, and nucleotide mismatch, for nearly all eukaryotic organisms. DSBs can arise from the exposure to exogenous agents, such as ultraviolet (UV) light from the sun, ionizing radiation (IR), carcinogens that are inhaled or ingested, and certain genotoxic chemicals, as well as from some endogenous biological processes, including DNA replication stresses and reactive oxygen species (ROS) which is produced as a byproduct of normal cellular metabolism. Homologous recombination (HR) and nonhomologous end joining (NHEJ) are two independent pathways responsible for repairing DNA DSBs. Unrepaired or inappropriately repaired DSBs can cause gene insertions, deletions, duplications, translocations, and chromosomal rearrangements. The giant mutations in genes arising from impaired DSBs could activate oncogenes, inactivate tumor suppressors, or increase the genome instability and eventually give rise to cell death, tumorigenesis, tumor progression or aging-related disease. Like many other types of cancers, defects in DSBs repair and increased genome instability may contribute to the tumorigenesis and development of HCC. Therefore, it is of high significance for the depth study of the mechanisms of DSBs repair in HCC. This would provide us some better understanding of the tumorigenesis, development, and treatment of HCC.

Yu et al*.* showed that PARG inhibition could significantly suppress the development of HCC and PARG inhibitors could produce a synergistic antitumor efficacy with anti-PD-1 antibodies in HCC [5]. In their work, the authors found that the mRNA and protein level of PARG was markedly upregulated in HCC tissues and the upregulated PARG expression was associated with a poor prognosis. Also, the in vitro and in vivo studied demonstrated that the overexpression of PARG could contribute to growth and metastasis in HCC, whereas the knockdown of PARG inhibited growth and metastasis in HCC. Mechanistically, PARG could directly interact with DNA damage-binding protein 1 (DDB1) and dePARylate DDB1, thereby regulate the expression of DDB1 by promoting its autoubiquitination and thus increasing the stabilization of the c-Myc protein. Overall, the author’s study revealed the oncogenic effect of PARG in the development of HCC and a potential therapeutic option for treating HCC targeted PARG with anti-PD-1 inhibitors. Here, we want to validate and provide some additional evidence about the regulation role of PARG in DNA repair of HCC.

To characterize the PARylated proteome upon DNA damage, we first performed an immunoprecipitation (IP) assay coupled with liquid chromatography-mass spectrometry (LC–MS) analysis to identify proteins that are PARylated by PARP1. The LC–MS results indicated that 6 peptides of DDB1 were detected (6.62% coverage) under the condition of X-Ray treatment, but no peptide was detected in the un-treatment group (Fig. 1a). We further verified the PARylation of DDB1 is dependent on DNA damage using Af1521 macrodomain (PAR/MAR) affinity resins in Hep3B HCC cell line (The detailed description of methods was presented in Supplementary Materials and Methods, Fig. 1b). We also performed an IP assay by using DDB1 antibody to confirm that the DDB1 was PARylated in response to DSBs indeed, but not bound by PAR chains in Hep3B HCC cell line (Fig. 1b). Moreover, it is reported that DDB1 could interact with PARP1 upon UV damage and DDB1 was a target for PARP-mediated PARylation. Taken together, we have revealed that the PARylation of DDB1 was largely dependent on DSBs in HCC, which was consistent with the author’s and previous studies.

**Fig. 1:**
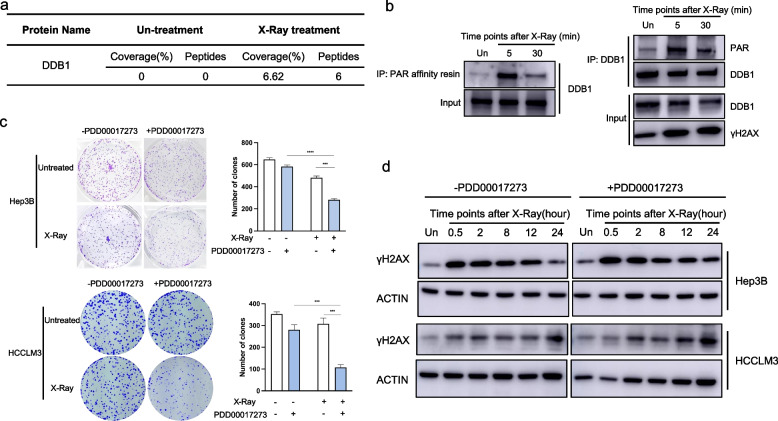
DDB1 is PARylated in response to DNA damage and the inhibition of PARG impairs DSBs repair in HCC. **a** Coverage and peptides number of PARylated DDB1 detected by LC–MS. **b** The Hep3B cells were lysed and immunoprecipitated with Af1521 macrodomain (PAR/MAR) affinity resins or an anti-DDB1 antibody followed by western blotting with an anti-DDB1 antibody or an anti-PAR antibody at the indicated time points after X-Ray treatment (4 Gy). **c** Colony-formation assays were applied to assess the survival of Hep3B and HCCLM3 cells treated with X-Ray (1 Gy), with PARG inhibitor (1 μM). **d** Western blotting assays for the effect of PARG inhibition (2 μM PDD00017273) on the γH2AX expression level in Hep3B and HCCLM3 cells at the indicated time points after X-Ray treatment (4 Gy)

The endogenous PARG levels of different HCC cell lines using quantitative RT-PCR and western blot indicated that PARG was highly expressed in Hep3B and HCCLM3 HCC cell lines [5]. To further confirm the functional role of PARG in HCC, we performed clonogenic assay and detected the expression level of phosphorylated histone H2AX (γH2AX), a biomarker of DSBs, in Hep3B and HCCLM3 HCC cell lines under the treatment of a well-known PARG inhibitor (PDD00017273) and X-Ray. Blocking PARG enzymatic activity using PDD00017273 sensitized Hep3B and HCCLM3 cells to DSBs induced by X-Ray, indicating that PARG is a critical factor for repairing DSBs (The detailed description of methods was presented in Supplementary Materials and Methods, Fig. 1c). By comparing the kinetics of γH2AX induced by X-Ray between control group and PARG inhibitor treated group, we found the inhibition of PARG caused a significant increase of the γH2AX level at 24 h after X-Ray treatment (Fig. 1d), which implied PARG inhibitor inhibited the clearance of γH2AX. Overall, these results indicated that inhibition of PARG could lead to decreased DSBs repair in HCC cells.

In response to DNA damage, the degradation of PAR chain (dePARylation) is indispensable for the proper DNA repair in cells. PARG is the primary enzyme involved in the dePARylation process of DNA repair. It was reported PARG could regulate DNA damage repair in different cancer cells and mouse cells, but the role of PARG in DSBs repair in HCC still needed to be further explored. Our study found that DDB1 was PARylated in response to DNA damage by using LC–MS, and we also confirmed that the PARylation of DDB1 was dependent on DSBs in HCC cells by using IP*.* Our results suggested that the inhibition of PARG impaired DSBs repair in HCC cells based on the clonogenic assay and the clearance of γH2AX, which provided additional mechanistic explanations for the impaired liver tumorigenesis of PARG inhibition. The indispensable role of PARG in DSBs repair in HCC provide additional evidence of targeting PARG for the HCC therapy.

## Supplementary Information

**Additional file 1.**

## Data Availability

Not applicable.
