# Supplementary material for: The inhibition of PARG attenuates DNA repair in hepatocellular carcinoma
Source: Mol Biomed. 2023 Jan 31;4:3. doi: 10.1186/s43556-023-00114-6 (PMC9889579; doi:10.1186/s43556-023-00114-6)
Supplement: Supplementary file 1 — Additional file 1. [file 43556_2023_114_MOESM1_ESM.docx]

# The inhibition of PARG attenuates DNA repair in hepatocellular carcinoma

Longpo Geng^1,#^, Yaling Sun^2,#^, Mingming Zhu^1,#^, Hongda An^1^, Yunzheng Li^1^, Yuanxiang Lao^1^, Yongli Zhang^3,*^, Binghua Li^1,*^, Jie Ni^1,*^, Zhu Xu^1,*^

^1^ Department of Hepatobiliary Surgery, The Affiliated Drum Tower Hospital of Nanjing University Medical School, Nanjing 210008, China

^2^ Department of Radiation Oncology, The Third Affiliated Hospital of Sun Yat-Sen University, Guangzhou 510630, China

^3^ Department of Gynecology of Shanghai First Maternity & Infant Hospital, Tongji University School of Medicine, Shanghai 201204, China

**^#^Equal contribution**

* **Corresponding author:** [xuzhu598@126.com](mailto:xuzhu598@126.com) (Z. Xu), [nijie@glyy.com](mailto:nijie@glyy.com) (J. Ni), [lbhnju@163.com](mailto:lbhnju@163.com) (B. Li), [doctorzhang2@163.com](mailto:doctorzhang2@163.com) (Y. Zhang)

**Supplementary Materials and Methods**

**Cell culture, reagents, and antibodies**

Human HCC cell lines (Hep3B and HCCLM3) were cultured in Dulbecco’s Modified Eagle Medium (DMEM) supplemented with 10% (v/v) of fetal bovine serum (FBS), 100 U/mL Penicillin and 100 µg/mL streptomycin. The cells were maintained at 37 °C in a humidified incubator with 5% CO2. PDD00017273 was purchased from MCE (#HY-108360). Af1521 macrodomain (PAR/MAR) affinity resins was purchased from Tulip BioLabs (#2302). DDB1 (#A5022) and γH2AX (#AP0099) antibodies were purchased from Abclonal. PAR antibody was purchased from Enzo life sciences (ALX-804-220-R100).

**Co-immunoprecipitation**

Cells were treated with X-Ray and harvested at the indicated time points. The collected cells were lysed with lysis buffer (20 mM HEPES pH 8.0, 0.2 mM EDTA, 10% glycerol, 150 mM NaCl, 1% NP40). The lysate was incubated on ice for 10 min, followed by sonication on ice at 50% duty for 5 s, and the lysate was then centrifuged at 12000 rpm for 30 min at 4◦C. The supernatant was collected for preclearing with Protein A+G Magnetic Beads (Beyotime, #P2108) and IgG antibody for 1 hour at 4◦C. The supernatant was collected and added with antibodies at concentrations as the suppliers suggested. After overnight incubation, Protein A+G Magnetic Beads were added to the lysate followed by rotating at 4◦C for 1 h. After washing 3 times with lysis buffer, 2× sample buffer was added and boiled for 10 min. Then the supernatant was collected for further western blotting analysis.

**Clonogenic assay**

Cells were pretreated with 1 μM PARG inhibitor in 6-well plates for 24 h. Then cells were treated with X-Ray at a dose of 1 Gy. On day 10 post the X-Ray treatment, cells were stained with commassie regent (0.25% Commassie, 50% methanol and 10% acetic acid) and colonies with at least 50 cells were counted.
